# *Morchella importuna* Flavones Improve Intestinal Integrity in Dextran Sulfate Sodium-Challenged Mice

**DOI:** 10.3389/fmicb.2021.742033

**Published:** 2021-09-06

**Authors:** Yingyin Xu, Liyuan Xie, Jie Tang, Xiaolan He, Zhiyuan Zhang, Ying Chen, Jie Zhou, Bingcheng Gan, Weihong Peng

**Affiliations:** ^1^National-Local Joint Engineering Laboratory of Breeding and Cultivation of Edible and Medicinal Fungi, Institute of Agricultural Resources and Environment, Sichuan Academy of Agricultural Sciences, Chengdu, China; ^2^Institute of Urban Agriculture, Chinese Academy of Agricultural Sciences, Chengdu, China

**Keywords:** *Morchella importuna*, intestinal barrier function, intestinal microbiota, inflammatory responses, C57BL/6 mice

## Abstract

*Morchella importuna*, as an edible fungus, has various health benefits. However, the effects of *M. importuna* on intestinal health are rarely investigated. Hence, this study aims to ascertain the influences of flavones from the fruiting bodies of *M. importuna* (hereinafter abbreviated as MIF) on dextran sulfate sodium (DSS)-induced damage to intestinal epithelial barrier in C57BL/6J mice. In this (14-day) study, 144 C57BL/6J mice were divided into four groups: (1) Control; (2) DSS treatment; (3) DSS treatment + 100 mg/kg MIF (LMIF); (4) DSS treatment + 200 mg/kg MIF (HMIF). On days 8-14, mice in the challenged groups were challenged with 3.5% DSS, while the control group received an equal volume of normal saline. Then, serum and intestinal samples were obtained from all mice. The results showed that MIF ingestion enhanced intestinal integrity in DSS-challenged mice, as evinced by the elevated (*p* < 0.05) abundances of occludin, claudin-1, and zonula occludens-1 proteins. Meanwhile, MIF ingestion reduced (*p* < 0.05) the colonic interleukin-1β (IL-1β), tumor necrosis factor-α (TNF-α), and interferon-γ (IFN-γ) concentrations and increased the superoxide dismutase and catalase activities and Shannon and Simpson indices in DSS-challenged mice. Moreover, MIF ingestion reduced (*p* < 0.05) the abundance of phospho-nuclear factor (NF)-κB and increased the abundance of phospho-Nrf2 in DSS-challenged mice. Taken together, MIF protects against intestinal barrier injury in C57BL/6J mice *via* a mechanism that involves inhibiting NF-κB activation and promoting Nrf2 activation, as well as regulating intestinal microbiota.

## Introduction

The intestinal epithelial barrier is a single layer of cells lining the gut that comprises the apical cell membrane and intercellular tight junctions of intestinal epithelial cells (Ulluwishewa et al., 2011; Peterson and Artis, 2014). It acts as a selective barrier that allows the absorption of nutrient substances while inhibiting the translocation of luminal pathogens (Halpern and Denning, 2015; Wang et al., 2015). Hence, treatments aimed at decreasing intestinal permeability contribute to improved health. Nowadays, dietary bioactive substances have been found to improve intestinal barrier function by strengthening the intestinal barrier, attenuating the inflammatory responses and modulating microbiota composition (Yang et al., 2012; Tian et al., 2019; Wan et al., 2020).

The *Morchella* mushroom, a type of edible ascomycetous mushroom, has a unique flavor, as well as a high nutritional value (Tietel and Masaphy, 2018). Interestingly, some bioactive compounds have been found in the *Morchella* mushroom fruiting body, such as polysaccharides, ergosterol derivatives, microthecin, and so on (Wang et al., 2019). Modern medical research demonstrated that the *Morchella* mushrooms confer antimicrobial, antioxidant, anti-inflammatory, and antitumor activities (Kim et al., 2011; Huang et al., 2012; Heleno et al., 2013). Therefore, the mature fruiting body of *Morchella* mushrooms have been used as a traditional herbal medicine in Asian countries, such as China, India, and Japan (Mau et al., 2004; Xiong et al., 2017). However, to the best of our knowledge, the ameliorative effects of *Morchella* mushrooms on intestinal barrier function have not been investigated and warrant exploration.

In the present work, the flavones from the fruiting bodies of *M. importuna* (MIF) were prepared. Then, the protective effects and possible mechanisms of MIF against the intestinal barrier injury *in vivo* were investigated using a sodium glucose sulfate [dextran sulfate sodium (DSS)]-challenged mouse model.

## Materials and Methods

### Preparation of the Fruiting Bodies of *Morchella importuna*

In this study, the MIF were collected from the experimental field of Sichuan Academy of Agricultural Sciences (Chengdu, China). The MIF were dried at 37°C, and then 200 g of MIF were immersed in proportions of 1:20 (w/v) in ethanol at 60°C for 6 h. After centrifugation at 6,000 × g for 15 min, the sediment was discarded. Thereafter, the resulting MIF was dried at 60°C and stored at −20°C before use.

### Preliminary Characterization of the Fruiting Bodies of *Morchella importuna*

The molecular weight distribution of MIF was determined by high-performance gel permeation chromatography (HP-GPC). The operating procedures were Waters 515, high-performance liquid chromatography equipped with laser detector (LS), and differential refractive index (DRI); Shodex OHpak series SB-806 gel chromatographic column (300 mm × 7.8 mm); column temperature 40°C ± 0.1°C. The mobile phase was 0.05 M NaH_2_PO_4_-NaH_2_PO_4_ buffer (pH 6.7, with 0.02% NaN_3_). The flow rate was 0.5 ml/min. The loading amount was 500 μl. Then, 0.05 M NaH_2_PO_4_-NaH_2_PO_4_ buffer (pH 6.7, with 0.02% NaN_3_) is used to dissolve polysaccharide standards with the molecular weights of 738, 5,800, 1.22 × 10^4^, 2.37 × 10^4^, 4.8 × 10^4^, 1.0 × 10^5^, 1.86 × 10^5^, 3.8 × 10^5^, and 8.53 × 10^5^ g/mol, respectively. After being filtered with 0.45-μm membrane, the determination was performed according to the above chromatographic conditions. According to the molecular weight and retention time of standards, the standard curve was drawn, and then the molecular weight was calculated according to the retention time.

### Animals, Management, and Diet

A total of 144 C57BL/6J mice (initial mass 18.02 ± 0.36 g), obtained from Dashuo Experimental Animal Co., Ltd. (Chengdu, China), were divided into four treatments with six pens per treatment (six mice per pen): (1) Control (fed a normal diet); (2) DSS treatment (fed a normal diet); (3) DSS treatment + 100 mg/kg MIF (LMIF; fed a normal diet + 100 mg/kg MIF); (4) DSS treatment + 200 mg/kg MIF (HMIF; fed a normal diet + 200 mg/kg MIF). On days 8–14, mice in the challenged groups were orally administered 3.5% DSS in drinking water, while other mice were administered normal saline (Bassaganya-Riera and Hontecillas, 2006). Moreover, all mice were individually caged under a controlled environment room.

### Slaughter and Sample Collection

At the end of the experiment, after 12-h starvation and ether anesthesia, blood samples from six mice with the average body weight in each group were collected, centrifuged at 1,500 × g (15 min) to obtain serum, and then stored at −20°C. Subsequently, the same mice were sacrificed, about 2-cm segments of the colon were isolated, gently flushed with normal saline, and then fixed in paraformaldehyde solution (4%) for morphological analysis. Finally, about 5-cm colonic tissues were collected and stored at −80°C until analyses.

### Serum Biochemical Analysis

The serum diamine oxidase (DAO) activity and D-lactate concentration were assessed using commercial kits purchased from Jiancheng Bioengineering Institute (Nanjing, China). All measurements were performed according to the manufacturer’s instructions.

### Intestinal Morphology Analysis

After a 48-h fixation, the colonic segments were dehydrated using a graded series of alcohol and cleaned with xylene, embedded in paraffin, cut into cross sections of 5-μm thickness, and then stained with H&E (Fang et al., 2017). Then, the villus height and crypt depth were measured, and the ratio of villus height to crypt depth (VCR) was calculated from the value obtained above.

### Intestinal Cytokine Concentration Determinations

The colonic mucosa was homogenized with normal saline (1:9), and the homogenate was centrifuged at 1,500 × g (15 min) to attain supernatant. Then, the concentrations of interleukin-1β (IL-1β), IL-6, IL-10, tumor necrosis factor-α (TNF-α), and interferon-γ (IFN-γ) in the colonic mucosal supernatant were assayed by ELISA kits (Zhuo Cai Biotechnology Co., Ltd., Shanghai, China).

### Intestinal Antioxidant Capacity Measurements

Superoxide dismutase (SOD) activity, catalase (CAT) activity, malondialdehyde (MDA) content, and total antioxidant capacity (T-AOC) in the colonic homogenates were measured. Measurements were performed by the spectrophotometric method using commercially available kits (Nanjing Jiancheng Bioengineering Institute).

### Intestinal Microbiota Analysis

Total gDNA from digesta samples was extracted using a Stool DNA Isolation Kit (Tiangen Biotech Co., Ltd., Beijing, China), following the manufacturer’s directions. The genes of bacterial 16S rRNA in the region of V4 were amplified by using PCR with primers (515F/806R). The PCR products were subjected to electrophoresis on 2% agarose gel, and the mixed PCR products were purified with AxyPrep DNA Gel Extraction Kit (Axygen Biosciences, Union City, CA, United States) for sequencing on an Illumina MiSeq system. All 16S rRNA gene sequencing data were saved in the National Center for Biotechnology Information and can be accessed in the Short Read Archive under the accession number PRJNA679459.^1^

Quality filtering on the raw reads was performed under specific filtering conditions to obtain the high-quality clean reads according to the Cutadapt quality-controlled process (Martin, 2011). The reads were compared with the reference database using UCHIME algorithm (Edgar et al., 2011), to detect chimera sequences, and then removed to get the clean reads (Haas et al., 2011). Clustered into operational taxonomic units (OTUs) utilizing Uparse v7.0.1001 at 97% sequence similarity (Edgar, 2013). Species annotation was carried out on the OTU representative sequences. For colonic bacteria, α-diversity index was assessed using QIIME 1.7.0. Principal coordinate analysis (PCoA) tools in R language were used for PCoA.

### Western Blot Assay

Protein samples were extracted from colonic tissues using lysis buffer (Beyotime Institute of Biotechnology, Shanghai, China). The lysates were centrifuged at 12,000 × g for 10 min at 4°C, and the supernatant was collected. A bicinchoninic acid (BCA) protein assay kit (Beyotime Institute of Biotechnology) was used to determine the protein concentration in the supernatant. Thereafter, 30 μg of protein extractions were separated by 10% sodium dodecyl sulfate (SDS)–polyacrylamide gel electrophoresis (PAGE) and then transferred to a polyvinyldifluoride (PVDF) membrane (Merck Millipore Ltd., Tullagreen, Ireland) using wet Trans-Blot System (Bio-Rad Laboratories, Inc., Hercules, CA, United States). After blocking with Tris-buffered saline Tween 20 (TBS/T) containing 5% bovine serum albumin (BSA) at room temperature for 1 h, the membranes were incubated with primary antibodies at 4°C overnight against phospho-Nrf2 (Sigma-Aldrich, St. Louis, MO, United States), Keap1 (Sigma-Aldrich), heme oxygenase-1 (HO-1; Sigma-Aldrich), NAD(P)H dehydrogenase (quinone 1) (NQO-1; Sigma-Aldrich), occludin (Sigma-Aldrich), claudin-1 (Proteintech Group, Inc., Wuhan, China), zonula occludens-1 (ZO-1; Sigma-Aldrich), ZO-2 (Sigma-Aldrich), Toll-like receptor 4 (TLR4; Proteintech Group, Inc.), MyD88 (Proteintech Group, Inc.), IL-1 receptor-associated kinase 1 (IRAK1; Proteintech Group, Inc.), TNF receptor-associated factor 6 (TRAF6; Proteintech Group, Inc.), phospho-NF-κB (Proteintech Group, Inc.), or β-actin (Proteintech Group, Inc.). The polyvinylidene fluoride (PVDF) membranes were washed thrice with TBS/T, then incubated with second antibodies at room temperature for 2 h, and washed thrice with TBS/T again. BeyoECL Moon (Beyotime Institute of Biotechnology) was used to visualize signals. The Image Lab software (Bio-Rad Laboratories, Inc.) was utilized to quantify protein abundance.

### Statistical Analysis

Individual rat was used as the experimental unit, and all data were analyzed by SPSS 20.0 (SPSS, Inc., Chicago, IL, United States). Statistical differences between groups were determined by Student’s *t*-test, while among groups, differences were determined by Tukey’s multiple-range test. Results were presented as means ± standard deviations. Differences were taken to indicate significance when *p* < 0.05.

## Results

### Molecular Weight and Its Distribution of *Morchella importuna* Flavones

From the results of HP-GPC detection, the mass average molar mass (Mw) of MIF was 6.666 × 10^5^ g/mol, the number average Molecular Weight (Mn) was 6.118 × 10^5^ g/mol, and the D value (Mw/Mn) was 1.09. The dispersity ratio was close to 1, and the molecular weight distribution was narrow, indicating that the MIF was relatively pure (Table 1).

**TABLE 1:** Molecular weight and its distribution of *Morchella importuna* flavones.

Item	MIF
Mw, g/moL	6.666 × 10^5^
Mn, g/moL	6.118 × 10^5^
Mw/Mn	1.09
**Molecular weight distribution, %**	
500000.0-522000.0 g/moL	5.30
522000.0-558000.0 g/moL	44.80
558000.0-805000.0 g/moL	37.20
805000.0-1170000.0 g/moL	7.20
1170000.0-2012949.0 g/moL	5.60

*MIF, fruiting bodies of M. importuna.*

### Serum Indices

DSS challenge enhanced (*p* < 0.05) the DAO activity and increased the concentration of D-lactate in C57BL/6J mice (Table 2). Dietary 200 mg/kg MIF inclusion reduced (*p* < 0.05) the serum D-lactate concentration in DSS-challenged mice.

**TABLE 2:** Effects of *Morchella importuna* flavones on the serum DAO activity and D-lactate concentration in DSS-challenged mice.

Item	Treatment^†^			
	CON	DSS	DSS + LMIF	DSS + HMIF
DAO, U/L	10.15 ± 3.33	15.86 ± 4.92*	11.15 ± 4.87	11.12 ± 2.98*
*D*-Lactate, pg/ml	14.91 ± 3.71	24.49 ± 5.28**	16.29 ± 3.84	11.72 ± 2.47**

*p < 0.05 or **p < 0.01.

*^†^CON, control; DSS, dextran sulfate sodium (DSS) treatment; DSS + LMIF, DSS treatment + 100 mg/kg fruiting bodies of M. importuna (MIF); DSS + HMIF, DSS treatment + 200 mg/kg MIF. DAO, diamine oxidase.*

### Intestinal Morphology

**FIGURE 1:**
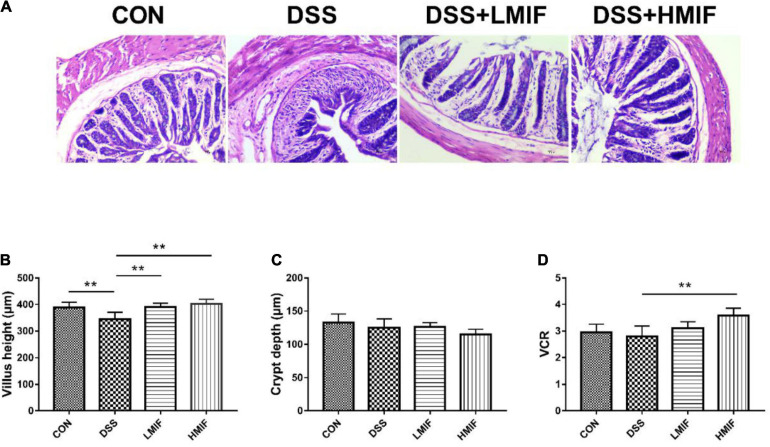
Effects of *Morchella importuna* flavones on colonic morphologies in dextran sulfate sodium (DSS)-challenged mice. **(A)** Colonic morphological image; **(B)** villus height; **(C)** crypt depth; **(D)** ratio of villus height to crypt depth (VCR). CON, control; DSS, DSS treatment; LMIF, DSS treatment + 100 mg/kg fruiting bodies of *Morchella importuna* (MIF); HMIF, DSS treatment + 200 mg/kg MIF. **p < 0.01.

Relative to the control mice, DSS challenge was found to reduce (*p* < 0.05) the colonic villus height without affecting crypt depth and VCR (Figure 1). Between the DSS-challenged mice, 100 and 200 mg/kg MIF supplementation increased (*p* < 0.05) the colonic villus height, and 200 mg/kg MIF supplementation additionally increased colonic VCR.

### Intestinal Antioxidant Capacity

According to Table 3, it is found that DSS challenge decreased (*p* < 0.05) the SOD, CAT, and T-AOC activities and increased the MDA content in the colon of C57BL/6J mice. Supplementation with 100 and 200 mg/kg MIF increased (*p* < 0.05) the colonic SOD and CAT activities in DSS-challenged mice.

**TABLE 3:** Effects of *Morchella importuna* flavones on the colonic antioxidant capacity in DSS-challenged mice.

Item	Treatment^†^			
	CON	DSS	DSS + LMIF	DSS + HMIF
SOD, U/ml	145.20 ± 2.30	93.43 ± 4.69**	118.46 ± 5.54**	117.49 ± 2.37**
CAT, U/ml	89.34 ± 0.88	65.10 ± 4.16**	84.70 ± 3.26**	88.01 ± 1.53**
T-AOC, U/ml	10.49 ± 0.51	4.80 ± 0.60**	5.70 ± 0.35	4.67 ± 0.61
MDA, nmol/ml	3.59 ± 0.24	4.54 ± 0.32*	4.14 ± 0.27	4.08 ± 0.15

*p < 0.05 or **p < 0.01.

*^†^CON, control; DSS, dextran sulfate sodium (DSS) treatment; DSS + LMIF, DSS treatment + 100 mg/kg fruiting bodies of M. importuna (MIF); DSS + HMIF, DSS treatment + 200 mg/kg MIF. CAT, catalase; MDA, malondialdehyde; SOD, superoxide dismutase; T-AOC, total antioxidant capacity.*

### Intestinal Cytokine Concentration

Dietary 200 mg/kg MIF ingestion reduced (*p* < 0.05) the contents of the IL-1β, TNF-α, and IFN-γ and increased (*p* < 0.05) the IL-10 content in colonic mucosa of DSS-challenged mice (Table 4). Moreover, 100 mg/kg MIF supplementation increased (*p* < 0.05) the colonic mucosal IL-10 concentration in DSS-challenged mice.

**TABLE 4:** Effects of *Morchella importuna* flavones on the colonic cytokine concentrations in DSS-challenged mice.

Item	Treatment^†^			
1.	CON	DSS	DSS + LMIF	DSS + HMIF
IL-1β, pg/ml	8.77 ± 1.09	15.02 ± 2.50**	12.51 ± 1.43	10.01 ± 1.34**
IL-6, pg/ml	11.30 ± 1.77	19.53 ± 4.64**	14.95 ± 3.26	13.45 ± 2.41*
IL-10, pg/ml	55.74 ± 5.24	38.70 ± 3.74**	50.49 ± 2.88**	53.79 ± 4.72**
TNF-α, pg/ml	83.07 ± 5.20	117.79 ± 7.30**	108.86 ± 7.36	107.51 ± 6.86*
IFN-γ, pg/ml	49.61 ± 5.33	77.37 ± 9.97**	68.91 ± 8.16	65.15 ± 7.65*

*p < 0.05 or **p < 0.01.

*^†^CON, control; DSS, dextran sulfate sodium (DSS) treatment; DSS + LMIF, DSS treatment + 100 mg/kg fruiting bodies of M. importuna (MIF); DSS + HMIF, DSS treatment + 200 mg/kg MIF. IFN, interferon; IL, interleukin; TNF, tumor necrosis factor.*

### Tight Junction Protein Abundances

**FIGURE 2:**
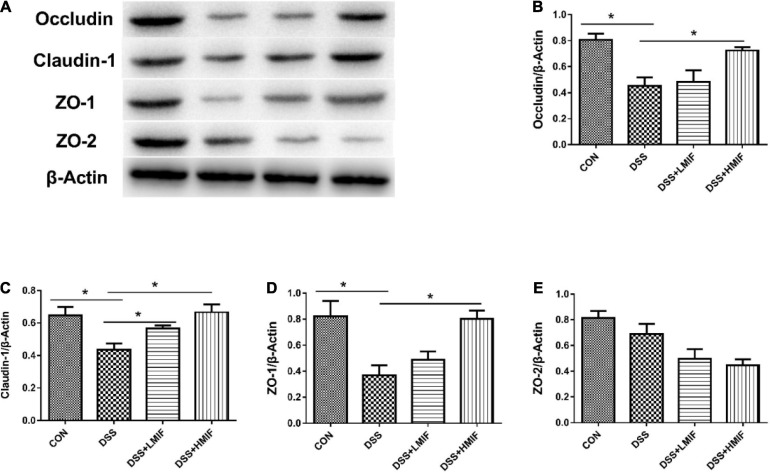
Effects of *Morchella importuna* flavones on the abundances of the colonic tight junction proteins in dextran sulfate sodium (DSS)-challenged mice. **(A)** Representative Western blot picture; **(B)** occludin; **(C)** claudin-1; **(D)** zonula occludens-1 (ZO-1); **(E)** ZO-2. CON, control; DSS, DSS treatment; LMIF, DSS treatment + 100 mg/kg fruiting bodies of *Morchella importuna* (MIF); HMIF, DSS treatment + 200 mg/kg MIF. *p < 0.05.

Figure 2 shows the effects of MIF on tight junction protein (occludin, claudin-1, ZO-1, and ZO-2) abundances in DSS-challenged mice. DSS challenge decreased (*p* < 0.05) the abundances of occludin, claudin-1, and ZO-1 proteins. Dietary supplementation with 100 and 200 mg/kg MIF elevated (*p* < 0.05) the abundance of claudin-1 protein, and 200 mg/kg MIF also increased (*p* < 0.05) the abundances of occludin and ZO-1 proteins in DSS-challenged mice.

### Nrf2 Pathway-Related Protein Abundances

**FIGURE 3:**
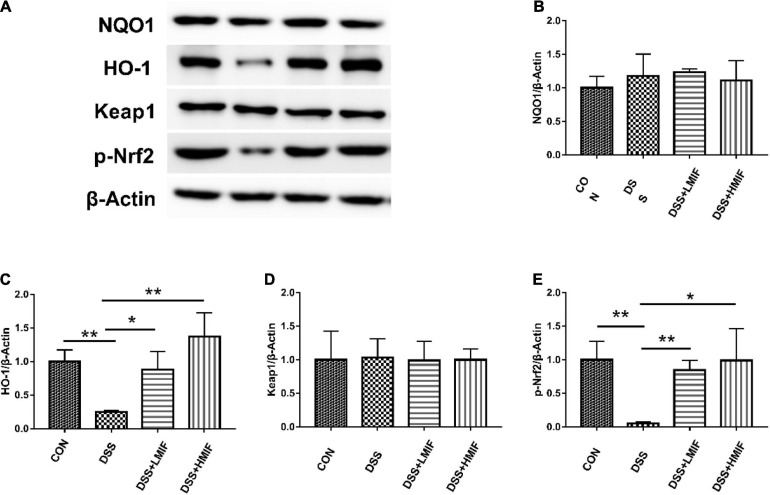
Effects of *Morchella importuna* flavones on the abundances of the colonic Nrf2 signaling pathway-related proteins in dextran sulfate sodium (DSS)-challenged mice. **(A)** Representative Western blot picture; **(B)** NAD(P)H dehydrogenase (quinone 1) (NQO-1); **(C)** heme oxygenase-1 (HO-1); **(D)** Keap1; **(E)** p-Nrf2. CON, control; DSS, DSS treatment; LMIF, DSS treatment + 100 mg/kg fruiting bodies of *Morchella importuna* (MIF); HMIF, DSS treatment + 200 mg/kg MIF. *p < 0.05 or **p < 0.01.

The differences in colonic Nrf2 pathway-related protein abundances among the four groups are shown in Figure 3. The colonic protein abundances of p-Nrf2 and HO-1 were lower in the DSS group (*p* < 0.05) than that in the control group. However, supplementation with 100 and 200 mg/kg MIF increased (*p* < 0.05) the colonic protein abundances of p-Nrf2 and HO-1 in DSS-challenged mice. Neither DSS nor MIF affected (*p* > 0.05) the Keap1 and NQO-1 protein abundances in C57BL/6J mice.

### NF-κB Pathway-Related Protein Abundances

**FIGURE 4:**
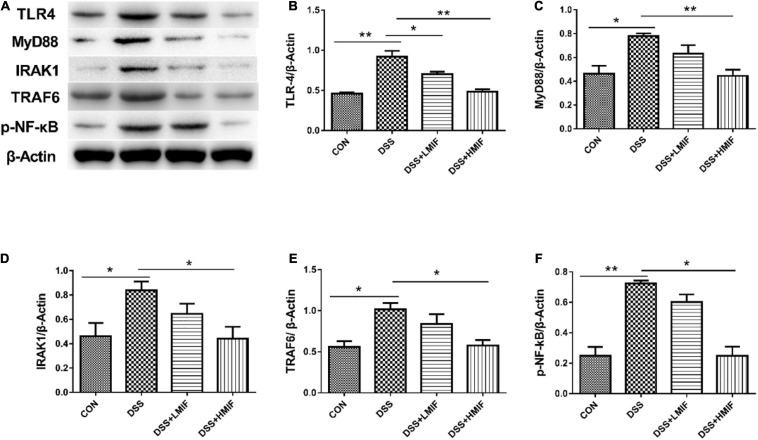
Effects of *Morchella importuna* flavones on the abundances of the colonic Toll-like receptor 4 (TLR4) signaling pathway-related proteins in dextran sulfate sodium (DSS)-challenged mice. **(A)** Representative Western blot picture; **(B)** TLR4; **(C)** MyD88; **(D)** IL-1 receptor-associated kinase 1 (IRAK1); **(E)** TNF receptor-associated factor 6 (TRAF6); **(F)** p-NF-κB p65. CON, control; DSS, DSS treatment; LMIF, DSS treatment + 100 mg/kg fruiting bodies of *Morchella importuna* (MIF); HMIF, DSS treatment + 200 mg/kg MIF. *p < 0.05 or **p < 0.01.

Figure 4 shows that the DSS challenge elevated (*p* < 0.05) the TLR4, MyD88, IRAK1, TRAF6, and p-NF-κB protein abundances, whereas supplementation with 200 mg/kg MIF reduced (*p* < 0.05) the TLR4, MyD88, IRAK1, TRAF6, and p-NF-κB p65 protein abundances in DSS-challenged mice. Moreover, 100 mg/kg MIF downregulated (*p* < 0.05) the TLR4 protein abundance in DSS-challenged mice.

### Intestinal Microbial Diversity

According to Table 5, it is found that DSS treatment decreased (*p* < 0.05) the Shannon index and Simpson index of bacteria in C57BL/6J mice. Supplementation with 200 mg/kg MIF increased (*p* < 0.05) the Shannon index and Simpson index of bacteria in DSS-challenged mice. Neither DSS nor MIF affected (*p* > 0.05) the Chao1 index or abundance-based coverage estimators (ACE) index of bacteria in C57BL/6J mice.

**TABLE 5:** Effects of ethanol extracts from *Morchella importuna* on the α-diversity indexes in the colon of DSS-challenged mice.

Item	Treatment^†^		
	CON	DSS	DSS + HMIF
Chao1 index	393.09 ± 30.64	371.89 ± 24.59	364.72 ± 12.33
ACE index	390.55 ± 23.95	374.08 ± 24.29	369.79 ± 13.76
Shannon index	6.27 ± 0.07	5.71 ± 0.20**	6.04 ± 0.04**
Simpson index	0.97 ± 0.00	0.94 ± 0.01**	0.96 ± 0.00**

**p < 0.01.

*^†^CON, control; DSS, dextran sulfate sodium (DSS) treatment; DSS + HMIF, DSS treatment + 200 mg/kg fruiting bodies of M. importuna (MIF).*

**FIGURE 5:**
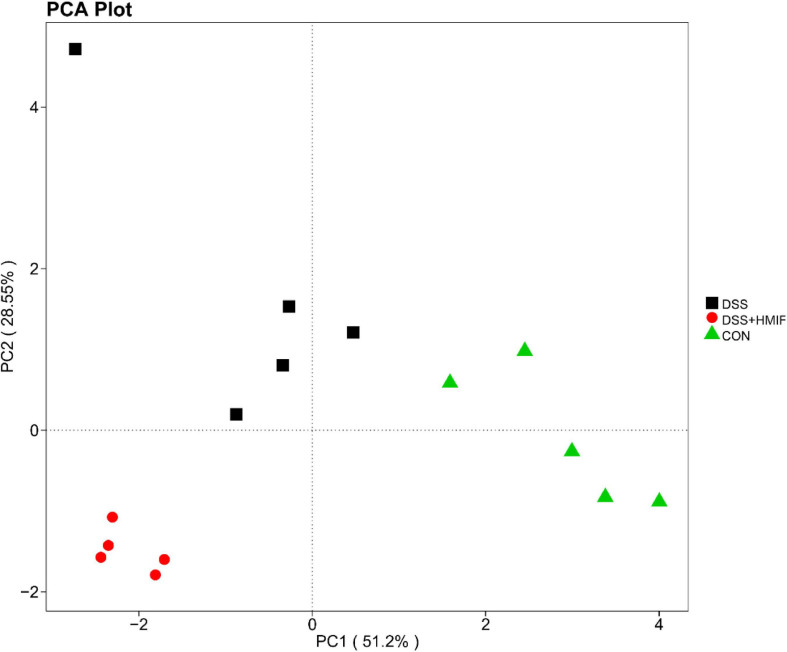
Comparison of the colonic microbiota composition among the three groups. A principal coordinate analysis (PCoA) was used to visualize the weighted UniFrac distances of the fecal samples from the C57BL/6J mice. CON, control; DSS, dextran sulfate sodium (DSS) treatment; HMIF, DSS treatment + 200 mg/kg fruiting bodies of *Morchella importuna* (MIF).

As shown in Figure 5, the PCoA revealed that microbial community was significantly altered after DSS challenge or MIF supplementation, with an evident separation (*p* < 0.05) among the three groups.

### Intestinal Microbiota Composition

**FIGURE 6:**
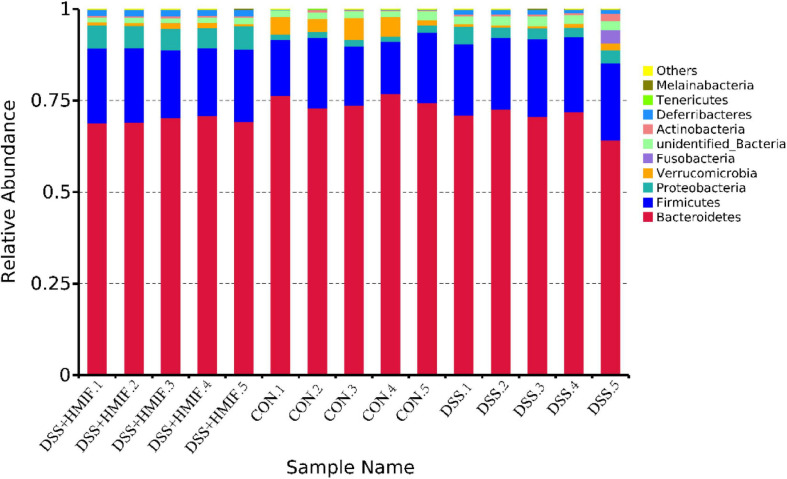
Relative abundances of the dominant bacteria at phylum level in the colon of C57BL/6J mice among the three groups. CON, control; DSS, dextran sulfate sodium (DSS) treatment; HMIF, DSS treatment + 200 mg/kg fruiting bodies of *Morchella importuna* (MIF).

The bacterial composition was assessed at different taxonomic levels (Figure 6 and Supplementary Table 1). At the phylum level, the dominant bacterial groups were *Bacteroidetes*, *Firmicutes*, and *Proteobacteria*; these were followed by the bacteria from phyla *Verrucomicrobia*, *Fusobacteria*, *Actinobacteria*, *Deferribacteres*, *Tenericutes*, and *Melainabacteria*. DSS challenge decreased (*p* < 0.05) the abundances of *Bacteroidetes* and *Verrucomicrobia*, increased (*p* < 0.05) the abundances of *Firmicutes*, *Proteobacteria*, *Deferribacteres*, and *Melainabacteria*. However, 200 mg/kg MIF supplementation increased (*p* < 0.05) the abundances of *Proteobacteria*, *Deferribacteres*, and *Melainabacteria*.

## Discussion

Villus height, crypt depth, and VCR serve as criteria that reflect gross intestinal morphology (Liu et al., 2008; Qin et al., 2018). At present, the DSS challenge decreased colonic villus height, which suggests that DSS caused acute damage to intestinal mucosa. MIF supplementation increased colonic villus height and VCR, which implies that MIF improved intestinal structure. The maintenance of intestinal integrity primarily depends on the tight junctions between the enterocytes. Tight junctions are composed of several tight junction proteins, such as occludin and claudins, as well as cytoplasmic ZOs (Anderson et al., 1993). Of them, occludin and claudins are considered to be the major integral membrane proteins forming continuous tight junction strands (Furuse et al., 1993; Furuse et al., 1998). Here, we found that MIF supplementation increased the abundances of occludin, claudin-1, and ZO-1 proteins in the colon of DSS-challenged mice, indicating that MIF improved the intestinal barrier integrity. Furthermore, intestinal integrity can be assessed by many markers, such as DAO activity and D-lactate concentration (Nielsen et al., 2011; Liu et al., 2012). Consistent with improved intestinal barrier function, MIF improved colonic barrier integrity in DSS-challenged mice, as evinced by decreased serum DAO activity and D-lactate concentration.

Intestinal antioxidant activity is closely related to intestinal health, which in turn is considered to be associated with intestinal structure (Jia et al., 2019). SOD and CAT are important antioxidant enzymes that can scavenge free radicals to defend against oxidative injury (Slavić et al., 2006; Lestaevel et al., 2009). We found lower SOD and CAT activities in DSS-challenged mice than in control mice, indicating that DSS challenge causes severe oxidative damage to the colon in mice. However, MIF attenuated the DSS-induced reduction of SOD and CAT activities in the colon, implying that MIF exerts a protective effect against intestinal oxidative damage caused by DSS challenge. The elevated antioxidant capacity was also supported by the expression of several critical antioxidant genes. Nrf2, one of the key transcription factors, plays a vital role in maintaining the activities of antioxidant enzymes (Cheng et al., 2015). The HO-1 is located downstream of the Nrf2 and acts as one of the key antioxidant enzymes (Han et al., 2017). In this study, MIF significantly elevated the protein levels of p-Nrf2 and HO-1 in the DSS-challenged mice, further indicating the antioxidant capacity of MIF in DSS-challenged mice. These results could determine that dietary MIF supplementation maintained the intestinal barrier function of mice under DSS challenge, to some extent, by enhancing intestinal antioxidant ability.

The unsettled balance between anti- and pro-inflammatory cytokines has been found to induce intestinal inflammatory injury in the DSS-challenged mice (Choi et al., 2017; Yin et al., 2020). In this study, MIF treatment inhibited inflammatory responses as evinced by decreasing pro-inflammatory cytokine (IL-1β, TNF-α, and IFN-γ) concentrations in the colon following DSS treatment. Contrary to the aforementioned cytokines, IL-10, as an anti-inflammatory cytokine, has been demonstrated to protect colonic inflammatory injury (Hasnain et al., 2013). Interestingly, MIF treatment also elevated the IL-10 concentration in the colon after DSS challenge. These results suggest that the beneficial effects of MIF against DSS-induced intestinal inflammatory injury were related to the regulation of the production of pro-inflammatory and anti-inflammatory cytokines. To elucidate the molecular mechanisms by which MIF attenuates intestinal inflammatory responses, we investigated the TLR4 signaling pathway-related protein expression.

Activation of TLR4 signaling pathway plays an important role in defensive responses against invading pathogens *via* triggering the secretion of pro-inflammatory cytokines (Wang et al., 2017). However, the aberrant activation of TLR4 signaling pathway elicits collateral host intestinal injury (Coll and O’Neill, 2010). In the present study, we observed that colonic protein abundances of TLR4 and its downstream signals, such as MyD88, IRAK1, and TRAF6, were reduced in MIF-treated DSS-challenged mice. NF-κB is a critical nuclear transcription factor downstream of the TLR4 signaling pathway that regulates the production of pro-inflammatory cytokines (Sabroe et al., 2008). The inactivation of NF-κB has been proven to be able to alleviate the severity of intestinal inflammatory injury (Kang et al., 2017; Wan et al., 2019). Here, the colonic protein abundance of p-NF-κB p65 in DSS-challenged mice was also decreased by MIF supplementation. These results suggest that MIF attenuates DSS-induced intestinal inflammatory injury *via* decreasing pro-inflammatory cytokine release through inhibiting the TLR4/NF-κB signaling pathway.

Although the exact pathogenesis of inflammatory bowel disease is complex, intestinal microbiota disorder is one of the most important observations (Zhai et al., 2019). As noted previously, the species, richness, and abundance of intestinal microbiota were markedly decreased in patients with inflammatory bowel disease (Zmora et al., 2019). In this study, we found that colonic microbiota in DSS-treated mice following MIF supplementation exhibit more diversity of evenness and richness than those in DSS-treated mice, as they have higher Shannon and Simpson indices. Low microbial diversity is often regarded as being associated with some infective intestinal disease, such as inflammatory bowel disease (Manichanh et al., 2006). Thus, the increase in microbial diversity induced by MIF may play a positive role in the colonic health of mice, which partly elucidates the alleviation of intestinal inflammatory injury in these mice. Furthermore, we found that MIF increased the abundances of *Proteobacteria*, *Deferribacteres*, and *Melainabacteria*, suggesting that these bacteria may play an essential role in MIF treatment of inflammatory bowel disease.

## Conclusion

To summarize, our findings indicate that MIF have beneficial effects on modulating intestinal barrier function and microbiota in DSS-challenged mice. The reduced inflammatory factor production and enhanced antioxidant capacity caused by MIF may be associated with inhibited NF-κB signaling pathway and activated Nrf2 signaling pathway, respectively. These results offer a molecular basis for the potential contribution of MIF to the prevention of intestinal barrier injury.

## Data Availability Statement

The datasets presented in this study can be found in online repositories. The names of the repository/repositories and accession number(s) can be found in the article/Supplementary Material.

## Ethics Statement

The animal study was reviewed and approved by the Animal Care and Use Committee of Sichuan Academy of Agricultural Sciences (Chengdu, China).

## Author Contributions

BG and WP conceived this study. YX wrote the manuscript. LX, JT, XH, ZZ, YC, and JZ carried out the experiments and performed data analyses. All authors contributed to the article and approved the submitted version.

## Conflict of Interest

The authors declare that the research was conducted in the absence of any commercial or financial relationships that could be construed as a potential conflict of interest.

## Publisher’s Note

All claims expressed in this article are solely those of the authors and do not necessarily represent those of their affiliated organizations, or those of the publisher, the editors and the reviewers. Any product that may be evaluated in this article, or claim that may be made by its manufacturer, is not guaranteed or endorsed by the publisher.

## Footnotes

1
http://www.ncbi.nlm.nih.gov/bioproject/679459
