# Supplementary material for: Morchella importuna Flavones Improve Intestinal Integrity in Dextran Sulfate Sodium-Challenged Mice
Source: Front Microbiol. 2021 Sep 6;12:742033. doi: 10.3389/fmicb.2021.742033 (PMC8451270; doi:10.3389/fmicb.2021.742033)
Supplement: Supplementary file 1 [file Table_1.doc]

**Journal name:** Frontiers in Microbiology

**Manuscript Title:** *Morchella importuna* flavones improve intestinal integrity in dextran sulfate sodium-challenged mice

**The names of the authors:** Yingyin Xu^1^, Liyuan Xie^1^, Jie Tang^1^, Xiaolan He^1^, Zhiyuan Zhang^1^, Ying Chen^1^, Jie Zhou^1^, Bingcheng Gan^2*^ and Weihong Peng^1*^

**The affiliations and addresses of the authors:** ^1^National-Local Joint Engineering Laboratory of Breeding and Cultivation of Edible and Medicinal Fungi, Institute of Agricultural Resources and Environment, Sichuan Academy of Agricultural Sciences, Chengdu 610066, Sichuan, People’s Republic of China

^2^Institute of Urban Agriculture, Chinese Academy of Agricultural Sciences, Chengdu 610213, Sichuan, People’s Republic of China

**The e-mail address of the corresponding authors:**

Bingcheng Gan, [ganbingcheng@caas.cn](mailto:ganbingcheng@caas.cn); Weihong Peng, whpeng768@163.com

**Table 1 Relative abundance of top 10 phyla in the colon of mice in three treatments^†^**

| Item | Treatment^†^ | | |
| --- | --- | --- | --- |
|  | CON | DSS | DSS + HMIF |
| *Melainabacteria* | 0.00000837±0.0000187 | 0.0000711±0.0000434^*^ | 0.00102±0.000133^**^ |
| *Tenericutes* | 0.000837±0.000311 | 0.000477±0.000275 | 0.000268±0.00006344 |
| *Deferribacteres* | 0.00141±0.000242 | 0.0117±0.00193^**^ | 0.0168±0.00120^**^ |
| *Actinobacteria* | 0.00248±0.00208 | 0.00808±0.00611 | 0.00417±0.000300 |
| *Unidentified_Bacteria* | 0.0189±0.00305 | 0.0244±0.00147^**^ | 0.0151±0.00170^**^ |
| *Verrucomicrobia* | 0.0422±0.0178 | 0.00981±0.00554^*^ | 0.00972±0.00466 |
| *Proteobacteria* | 0.0168±0.00206 | 0.0332±0.00881^*^ | 0.0603±0.00384^**^ |
| *Firmicutes* | 0.168±0.0228 | 0.204±0.00769^*^ | 0.195±0.00914 |
| *Bacteroidetes* | 0.749±0.0171 | 0.701±0.0337^*^ | 0.697±0.00831 |

^*^*P* < 0.05 or ^**^*P* < 0.01.

^†^CON, control; DSS, dextran sulfate sodium (DSS) treatment; DSS + HMIF, DSS treatment + 200 mg/kg fruiting bodies of *M. importuna* (MIF).
